# Vasopressin–aquaporin-2 pathway: recent advances in understanding water balance disorders

**DOI:** 10.12688/f1000research.16654.1

**Published:** 2019-02-04

**Authors:** Marianna Ranieri, Annarita Di Mise, Grazia Tamma, Giovanna Valenti

**Affiliations:** 1 Department of Biosciences, Biotechnologies and Biopharmaceutics, University of Bari, Bari, Italy, 70125, Italy; 2 Istituto Nazionale di Biostrutture e Biosistemi, Rome, Roma, Italy, 00136, Italy; 3 Center of Excellence in Comparative Genomics (CEGBA), University of Bari, Bari, Italy, 70125, Italy

**Keywords:** Vasopressin, AQP2, NDI, SIADH, NSIAD, ADPKD

## Abstract

The alteration of water balance and related disorders has emerged as being strictly linked to the state of activation of the vasopressin–aquaporin-2 ****(vasopressin–AQP2) pathway. The lack of responsiveness of the kidney to the vasopressin action impairs its ability to concentrate the urine, resulting in polyuria, polydipsia, and risk of severe dehydration for patients. Conversely, non-osmotic release of vasopressin is associated with an increase in water permeability in the renal collecting duct, producing water retention and increasing the circulatory blood volume. This review highlights some of the new insights and recent advances in therapeutic intervention targeting the dysfunctions in the vasopressin–AQP2 pathway causing diseases characterized by water balance disorders such as congenital nephrogenic diabetes insipidus, syndrome of inappropriate antidiuretic hormone secretion, nephrogenic syndrome of inappropriate antidiuresis, and autosomal dominant polycystic kidney disease. The recent clinical data suggest that targeting the vasopressin–AQP2 axis can provide therapeutic benefits in patients with water balance disorders.

## Introduction

The maintenance of water balance is essential for all physiological processes and is critically dependent on water intake via thirst and water output in the kidney under the control of the antidiuretic hormone vasopressin. An increase in plasma osmolality, sensed by osmoreceptors situated in the brain, represents the most important input to cause thirst and stimulation of vasopressin release. Vasopressin is secreted into the circulation by the posterior pituitary gland in response to an increase in serum osmolality or a decrease in blood volume. In the kidney, vasopressin binds to the type 2 vasopressin receptor (V2R) and increases osmotic water transport through the regulation of the aquaporin-2 (AQP2) water channel localized in the kidney connecting tubules and collecting ducts ^1, 2^. V2R is a G-protein-coupled receptor (GPCR) localized at the basolateral plasma membrane of the principal cells of the kidney collecting duct. A recent transcriptome study of 14 microdissected nephron segments of rat kidney demonstrated the V2R mRNA expression from the medullary thick ascending limb to the inner medullary collecting duct ^3^. Upon binding of vasopressin to V2R, a G _s_ protein is activated, leading to stimulation of adenylyl cyclases, increase in intracellular cAMP, and activation of protein kinase A (PKA). The cAMP/PKA signal transduction cascade results in multiple phosphorylating events in the C-terminus of the water channel AQP2 regulating its trafficking and the water luminal permeability ^2, 4– 6^. Vasopressin also triggers increases in intracellular calcium required for AQP2 trafficking ^7, 8^.

Most of the effect of vasopressin is thought to be related to PKA-mediated phosphorylation currently explored by large-scale phosphoproteomics to identify regulated proteins’ downstream PKA activation ^9^. On the other hand, several proteins participating in the control of cAMP-dependent AQP2 trafficking, including SNAREs, annexin-2, hsc70, A-kinase anchoring proteins (AKAPs), and small GTPases of the Rho family proteins controlling cytoskeletal dynamics, have been identified ^10– 18^. In addition to phosphorylation, AQP2 undergoes different regulated post-translational modifications, such as ubiquitination and glutathionylation, which are likely to be fundamental for controlling AQP2 cellular localization, stability, and function ^19– 23^.

Besides short-term regulation of AQP2 trafficking, vasopressin regulates the total amount of the water channel within the cell and alters the protein half-life of AQP2 ^24– 29^. Alterations in AQP2 abundance as well as defects in vasopressin signaling in the renal collecting duct can seriously compromise renal function and the maintenance of water balance in the body.

This review highlights some of the new insights and recent advances in targeting the vasopressin–AQP2 pathway in some relevant diseases associated with water balance disorders, such as congenital nephrogenic diabetes insipidus (NDI), idiopathic syndrome of inappropriate antidiuretic hormone secretion (SIADH), nephrogenic syndrome of inappropriate antidiuresis (NSIAD), and autosomal dominant polycystic kidney disease (ADPKD).

## Vasopressin–AQP2 pathway in water balance disorders

### Plasma copeptin as a surrogate marker of vasopressin secretion in renal disorders

Significant progress in studying the role of vasopressin in renal disorders came from the identification of copeptin, a stable surrogate marker of vasopressin secretion that is relatively easily measured ^30– 33^. Copeptin is a peptide corresponding to the COOH-terminal portion of pro-vasopressin and is co-secreted in equimolar amounts with vasopressin, representing a good biomarker for vasopressin ^34^. Indeed, it has been reported that plasma concentrations of copeptin correlate strongly in several clinical conditions ^32, 33, 35, 36^. In a large cross-sectional study, plasma copeptin and microalbuminuria positively correlated ^37^, and this correlation was also found to persist with a 16-year follow-up ^38^. The increase in urinary albumin excretion is likely to reflect not only glomerular damage but also systemic endothelial dysfunction and is consistent with the hypothesis that vasopressin induces urinary albumin excretion as previously reported in rats and humans ^39, 40^. Conversely, suppressing vasopressin by administering a V2R antagonist or by simply increasing water intake might be beneficial for renal function and diabetes ^39, 41^.

As shown previously, vasopressin levels are also increased in diabetic nephropathy characterized by dysregulation of water balance displaying water depletion as a consequence of osmotic diuresis due to glycosuria ^42^, probably to limit water loss. Of interest, in patients with type 2 diabetes, plasma copeptin was found to be associated with a faster decline in glomerular filtration rate (GFR) in two distinct studies ^43, 44^.

The role of vasopressin is particularly central in the pathogenesis of another severe disease, ADPKD, characterized by the expansion of renal cysts eventually leading to loss of renal function. Association of urinary copeptin with the severity of ADPKD was also recently demonstrated ^44^, suggesting that copeptin can represent a novel marker to predict renal prognosis in ADPKD. Copeptin levels are negatively associated with GFR, kidney size, and number of renal cysts ^33, 45, 46^. While ADPKD is the most advanced disease for the therapeutic use of vasopressin receptor blockade, this strategy is also currently being explored in chronic kidney diseases (CKDs).

In summary, the vasopressin–AQP2 system has a critical role in various stages of CKD ^47– 49^ and in several kidney diseases ^46^, making this pathway very promising from a therapeutic perspective. Indeed, high water intake (particularly plain water) has been proven to be beneficial in preventing CKD progression in several studies (reviewed in 46), based on the expected suppression of vasopressin release that can be easily monitored by plasma copeptin measurements. On the other hand, the association between total fluid intake (excluding water intake) and change in kidney function requires additional prospective studies, since other data do not support the hypothesis that total fluid consumption (excluding water intake) protects against CKD ^50^; therefore, additional study with prospective evaluation of kidney function is required.

### Targeting defective vasopressin–AQP2 pathway in nephrogenic diabetes insipidus

NDI is a disease characterized by the kidney’s inability to concentrate urine despite elevated concentrations of vasopressin. The congenital form of NDI can be due to mutation of either the vasopressin receptor or AQP2 and is associated with marked polyuria, polydipsia, and electrolyte imbalance with a constant risk of severe dehydration for patients ^51, 52^. Most of the forms of NDI are due to non-functional V2R (X-linked NDI) ^53^. X-linked NDI accounts for 90% of cases of congenital NDI and occurs with a frequency of 4 to 8 per 1 million male live births. Autosomal NDI accounts for about 10% of the remaining cases ^54^.

NDI can also be acquired and is a frequent side effect of lithium therapy ^55^ or other drugs ^56– 60^. Hypokalemia or hypercalcemia with associated hypercalciuria also cause acquired NDI ^28, 61– 68^.

The year 2013 was the 100th anniversary of vasopressin treatment for central diabetes insipidus due to vasopressin hormone deficiency ^69^. Despite extensive research in the field, a real cure for NDI is still missing and treatment is rather symptomatic with a continuous supply of water and drugs such as ibuprofen and indomethacin in combination with hydrochlorothiazide, which however only partially (by 25% to 50%) reduce the abundant polyuria ^51, 68, 70^.

The therapeutic approaches under investigation regard the rescue of mutated V2R or AQP2, in case of defective trafficking of these proteins, or bypassing the V2R signaling, in case of inactive V2Rs, finalized to increasing the cell surface expression of AQP2 ^51, 71– 73^.

In patients with NDI due to V2R mutations or as a consequence of lithium therapy, AQP2 is in fact theoretically functioning, suggesting the possibility of increasing its apical membrane abundance independently from vasopressin or cAMP. Some studies suggest that this may be possible. Sildenafil, a phosphodiesterase inhibitor causing an increase in cyclic guanosine monophosphate, reduced polyuria in rats with lithium-induced NDI ^74^. Moreover, a child affected by X-linked NDI treated with sildenafil had a beneficial effect in decreasing urine volume and increased urine osmolality ^75^.

Another approach to bypass defective V2R was altering the cholesterol content of the apical membrane to increase AQP2 accumulation. To this end, different statins have been used. Statins are a family of 3-hydroxy-3-methylglutaryl-CoA reductase inhibitors commonly used to inhibit cholesterol biosynthesis. Besides this, *in vitro* studies showed that statins reduced endocytosis possibly by inhibiting isoprenylation of Rho GTPase, which leads to actin cytoskeletal reorganization during protein trafficking ^76^. Of note, in renal cells and *in vivo*, statins have been shown to promote AQP2 localization to the apical membrane ^77, 78^, a process known to require apical depolymerization of the actin network and Rho inhibition ^11, 12^.

Interestingly, a combination of two statins—fluvastatin and secretin—drastically reduced urine output by nearly 90% and doubled urine osmolality in mouse models of NDI in a mouse model ^79^. Statin treatment was also found to be beneficial in NDI patients with inactivating mutation of the V2R ^78^ and in patients under lithium therapy ^80^. Besides the effect in reducing urinary output in patients with NDI, the efficacy of the treatment with statins in increasing apical expression of AQP2 has been monitored by measuring the urinary excretion of AQP2 (u-AQP2), which was found to be increased in a time- and dose-dependent manner ^81^.

The most recent approach for treating congenital X-linked NDI is based on treatment with metformin, a drug used for the treatment of diabetes which is able to activate the adenosine monophosphate kinase (AMPK) ^82, 83^. In the renal inner medullary collecting duct, AMPK phosphorylates AQP2 and urea transporter UT-A1, resulting in water and urea reabsorption ^83^, and improves renal concentrating ability in X-linked NDI mice ^82^. Therefore, AMPK activation with metformin might represent an alternative therapeutic approach to treat X-linked NDI. More recently, it was shown that inhibition of AKAPs binding to PKA increases PKA activity and activates AQP2 channels in cortical collecting duct cells ^84^. In line with this finding, *in vivo* experiments revealed that the compound 3,3′-diamino-4,4′-dihydroxydiphenylmethane (FMP-API-1) induced AQP2 phosphorylation, trafficking, and water reabsorption to the same extent as vasopressin in the context of V2R inhibition. This study points to AKAP-PKA disruptors as a potential novel category of therapeutic drugs for congenital NDI ^84^.

Targeting selected GPCRs with agonists to increase cAMP has also been considered an option to treat NDI. Among the GPCRs expressed in the inner medulla collecting duct, activation of the calcitonin receptor caused an increase in cAMP and accumulation of AQP2 in the plasma membrane in LLCKP1 cells ^85^ and reduced urinary output in vasopressin-deficient Brattleboro rats ^79^. Other GPCRs whose activation was found to be associated with increased AQP2 expression are the angiotensin II AT1 receptor ^86^ and secretin receptor ^79, 87^. However, it has to be underlined that *in vivo* testing of this approach targeting GPCRs revealed that the effect is transient and this is probably due to desensitization of the receptors ^79, 85^. In parallel, four other GPCRs, the protein-coupled E-prostanoid receptors EP1–EP4, have been considered for their ability to increase cAMP and AQP2 in MDCK cells ^88^. Selective silencing of EP4 in mice resulted in reduced renal concentrating ability ^89^. Interestingly, in renal collecting duct cell models, EP4 activation increased AQP2 trafficking independently from cAMP elevation ^73, 90^, although the mechanisms responsible for EP4-induced stimulation of AQP2 trafficking are not yet clarified.

The calcium signaling pathway in the vasopressin response has also been considered a major target in the treatment of NDI. Specifically, activators of calcium signaling in collecting duct principal cells may represent a therapeutic strategy in NDI. In a recent seminal study, Uchida *et al*. demonstrated that activation of the calcium signal transducer Wnt5a, through a calcium/calmodulin/calcineurin signaling pathway, induced phosphorylation, trafficking, and mRNA expression of AQP2 in mpkCCDCl4 cells ^91^. Of note, in isolated cortical collecting ducts from a V2R-inhibited NDI mouse model, Wnt5a increased osmotic water permeability and urine osmolality, activating a different pathway of the vasopressin-induced cAMP elevation, involving a functional role of calcineurin/arachidonic acid known to induce vasopressin-like effects in mpkCCD cells. These new data point to calcineurin activators as possible drugs for the treatment of congenital NDI.

### Vasopressin–AQP2 pathway in syndrome of inappropriate antidiuretic hormone secretion and nephrogenic syndrome of inappropriate antidiuresis

SIADH may be considered an opposite disease with respect to NDI. Patients with SIADH have high levels of vasopressin even in the presence of low serum osmolality, leading to dilutional hyponatremia secondary to elevated renal water reabsorption ^92, 93^.

Hyponatremia is defined as a serum Na level below 135 mmol/L and is associated with an increase in mortality in hospitalized patients ^94^. In SIADH, high levels of vasopressin are present even when plasma osmolality reaches values that normally suppress vasopressin release and the resulting hyponatremia can be ascribed to a non-osmotic release of vasopressin ^95^. Nevertheless, a phenomenon called “vasopressin escape” allows the kidney to excrete free water through reduced expression of the gene encoding AQP2, thus increasing water excretion ^96, 97^. Interestingly, a recent study uncovered some signaling mechanisms that defend against hyponatremia in SIADH ^21^. A SIADH-like condition is frequently related to aging ^93^. SIADH has several major etiologies, including central nervous system and pulmonary disorders, tumors, and drugs, the last of which is related mainly to psychopharmacological treatment. An interesting observation is that treatment with antipsychotics is associated with elevated activity of calcineurin and enhanced vasopressin release, which may contribute to activation of AQP2 trafficking causing drug-induced SIADH. Moreover, cyclophosphamide, an anti-cancer drug, activates V2R and induces AQP2 expression in rat kidney despite the absence of vasopressin stimulation ^98^. This finding suggested the possibility of drug-induced NSIAD. The syndrome of inappropriate antidiuresis has been suggested as an alternative to SIADH, since a subgroup of patients with features of SIADH does not have high plasma vasopressin levels ^99^. Therefore, dilutional hyponatremia may result from either excessive AVP release or constitutive activation of V2R.

The use of antagonists of the vasopressin receptors (vaptans) represents the most direct treatment for hyponatremia in SIADH. In this respect, over the last 10 years, significant advances in the treatment of SIADH have been made ^100^.

Among vaptans, conivaptan represents the first vasopressin receptor blocker approved by the US Food and Drug Administration ^93^. Conivaptan is given intravenously and acts on both V1 and V2 vasopressin receptors, resulting in solute-free water diuresis (aquaresis). Tolvaptan instead is orally active and is a selective antagonist of the V2R. It has been shown to be effective and safe for up to 3 years ^101^. Both conivaptan and tolvaptan are approved for the treatment of hypervolemic hyponatremia in patients with heart failure or cirrhosis and of euvolemic hyponatremia in patients with SIADH. We recently demonstrated that tolvaptan prevents AQP2 trafficking and function in collecting duct principal cells and reduces AQP2 excretion in two patients with SIADH paralleled by normalization of plasma sodium concentration, clearly demonstrating the central role of AQP2 blockade in the aquaretic effect of tolvaptan ^102^. An alternative approach with broad potential clinical application is the identification of small-molecule inhibitors of aquaporins ^103^; however, so far, progress in the field has been disappointing.

As discussed, SIADH is characterized by hypersecretion of vasopressin. However, 10% to 20% of patients with inappropriate antidiuresis display low or undetectable vasopressin circulating levels. This condition was found to be associated with gain-of-function mutations of the V2R, first discovered in 2005 ^104^, and the associated disease was defined as NSIAD to distinguish it from SIADH. NSIAD is a very rare disease representing the mirror image of NDI; 21 cases have been reported to date and the majority of them come from five families. In the first two cases of NSIAD, the mutations of the V2R were found at residue R137.

In the first two cases of NSIAD, the mutations of the V2R gene ( *AVPR2*) result in changes in codon 137 from arginine to cysteine or leucine (R137C and R137L). Of note, whereas substitution of R137 to cysteine or leucine results in NSIAD, conversion of R137 to histidine (R137H) is a well-known loss-of-function mutation associated with NDI, leading to water loss and inability to concentrate urine ^105^. Thus, mutations of the same amino acid of the V2R, R137, can have opposite clinical outcomes. Other constitutively activating mutations causing NSIAD—namely F229V ^106^, I130N ^107^, and L312S ^108^—have been subsequently identified. Theoretically, NSIAD should be treated with a V2R inverse agonist; however, tolvaptan and satavaptan had no efficacy in patients carrying mutations of R137 ^109^. In contrast, both drugs have an effect on F229V, I130N mutants, and L312S *in vitro*, suggesting that they could be effective in patients carrying these mutations ^106– 108^.

### Targeting the vasopressin–AQP2 pathway in autosomal dominant polycystic kidney disease

ADPKD is an inherited disorder with an estimated frequency of 1 in 400 to 1,000 live births ^110^ and is characterized by the progressive growth of renal cysts causing disruption of renal architectures and eventually leading to end-stage renal disease. Despite high baseline vasopressin levels, patients frequently present defective urinary concentration, and this is probably due to a peripheral resistance to vasopressin ^111, 112^. Interestingly, the blockade of V2R using tolvaptan in patients with rapidly progressing ADPKD has been proven to slow cyst growth, supporting the involvement of the V2R pathway in ADPKD ^113, 114^.

The hereditary form of ADPKD is due to mutations in *PKD1* or *PKD2* genes, encoding for polycystin 1 (PC1) and polycystin 2 (PC2), respectively. PC1 is localized to the primary cilium and in cell–cell contacts, suggesting a role as an adhesion protein ^115^. PC2, instead, is a non-selective cation channel permeable to calcium, expressed in the endoplasmic reticulum and in primary cilium, where it forms a complex with PC1 and has a role in intracellular Ca ^2+^ homeostasis. At the cellular level, mutations in *PKD1* or *PKD2* are associated with a reduction in intracellular calcium, increase in cAMP, and constitutive activation of PKA, making the collecting duct principal cells under constant tonic effect of vasopressin. The disruption in calcium and cAMP signaling cascades activates pathways causing cell proliferation, increased fluid secretion, and interstitial inflammation ^116, 117^. Our recent *in vitro* study demonstrated that in human conditionally immortalized proximal tubular epithelial cells silenced for PKD1 (ciPTEC-PC1KD) or generated from a patient with ADPKD1 (ciPTEC-PC1Pt), selective activation of the calcium-sensing receptor increases cytosolic calcium, reduces intracellular cAMP and mTOR activity ^118^, and rescues defective ATP mitochondrial production ^119^, reversing the principal ADPKD dysregulations.

Currently, there is no cure for the disease; it is clinically manageable only through the control of its many complications, and the existing therapeutic approaches are rather supportive. Nevertheless, as mentioned, some drugs targeting the vasopressin–AQP2 pathway have been found to slow the progression of ADPKD in animal models and clinical trials ^120^. Tolvaptan has been tested on ADPKD patients with higher total kidney volume and was found to delay the progression of ADPKD, supporting the link between V2R signaling and ADPKD development ^113, 121^. Tolvaptan has been approved to delay the progression of ADPKD in Japan, Canada, and the European Union, and very recently the US Food and Drug Administration also approved tolvaptan as the first drug treatment to slow kidney function decline in adults at risk of rapidly progressing ADPKD.

## Conclusions and perspectives

**Figure 1.**
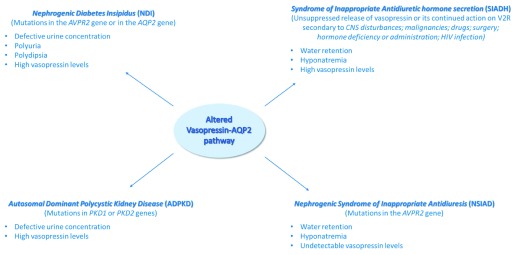
Targeting the vasopressin–AQP2 pathway in water balance disorders. Alterations of the vasopressin–AQP2 axis, causes of the disease, and principal effects observed in selected water balance disorders. AQP2, aquaporin-2; CNS, central nervous system; V2R, type 2 vasopressin receptor.

Over the past decade, interest in the vasopressin–AQP2 pathway has been renewed, mainly because of the availability of vaptans, the orally active vasopressin receptor antagonists, and of the use of copeptin as a surrogate marker of vasopressin. The dysregulation of the vasopressin–AQP2 system is clearly and tightly associated with specific diseases such as NDI, SIADH, NSIAD, and ADPKD, briefly discussed in this review ( Figure 1).

With regard to NDI, many target molecules for the treatment of congenital NDI have been proposed; however, no specific pharmacological drugs have yet reached clinical application. In the development of drugs for the treatment of congenital NDI, it is important to identify/design drugs that directly activate AQP2 without toxic effects and to pay attention to preserving the medullary osmotic gradient, representing the driving force for water reabsorption. In this scenario, the screening for calcineurin activators like Wnt5a is a potentially promising therapeutic strategy to develop novel drugs for the treatment of heritable NDI.

Conversely, for the management of opposite diseases such as SIADH, characterized by hyperactivation of the vasopressin–AQP2 pathway, as discussed in this review, the use of vaptans to block vasopressin receptors represents the main avenue for the direct treatment of hyponatremia. Although among vaptans tolvaptan is well tolerated, the design of new V2R blockers reducing the reported side effects should be encouraged. Moreover, co-targeting V2R and other GPCRs known to increase intracellular calcium might be a successful approach for ADPKD treatments.

Novel AQP-targeting therapies through modulation of microRNA (miRNA) function have recently been suggested (reviewed in 122). The possibility of using miRNA alone or in combination with other drugs to modulate the vasopressin–AQP2 pathway specifically provides new hints for AQP-based therapeutics ^27, 123^. In this respect, the recent identification of miR-132 as a first miRNA target which blocks vasopressin gene expression has opened new avenues for drug development ^124^.

## Abbreviations

ADPKD, autosomal dominant polycystic kidney disease; AKAP, A-kinase anchoring protein; AMPK, adenosine monophosphate kinase; AQP2, aquaporin-2; CKD, chronic kidney disease; GFR, glomerular filtration rate; GPCR, G-protein-coupled receptor; miRNA, microRNA; NDI, nephrogenic diabetes insipidus; NSIAD, nephrogenic syndrome of inappropriate antidiuresis; PKA, protein kinase A; SIADH, syndrome of inappropriate antidiuretic hormone secretion; V2R, type 2 vasopressin receptor
